# Using Point of Care Testing to estimate influenza vaccine effectiveness in the English primary care sentinel surveillance network

**DOI:** 10.1371/journal.pone.0248123

**Published:** 2021-03-11

**Authors:** Simon de Lusignan, Uy Hoang, Harshana Liyanage, Manasa Tripathy, Julian Sherlock, Mark Joy, Filipa Ferreira, Javier Diez-Domingo, Tristan Clark

**Affiliations:** 1 Nuffield Department of Primary Care Health Sciences, University of Oxford, Oxford, United Kingdom; 2 Vaccine Research Department, FISABIO-Public Health, Valencia, Spain; 3 Academic Unit of Clinical and Experimental Sciences, University of Southampton, Southampton, United Kingdom; Federal University of Sergipe, BRAZIL

## Abstract

**Introduction:**

Rapid Point of Care Testing (POCT) for influenza could be used to provide information on influenza vaccine effectiveness (IVE) as well as influencing clinical decision-making in primary care.

**Methods:**

We undertook a test negative case control study to estimate the overall and age-specific (6 months-17 years, 18–64 years, ≥65 years old) IVE against medically attended POCT-confirmed influenza. The study took place over the winter of 2019–2020 and was nested within twelve general practices that are part of the Oxford-Royal College of General Practitioners (RCGP) Research and Surveillance Centre (RSC), the English sentinel surveillance network.

**Results:**

648 POCT were conducted. 193 (29.7%) of those who were swabbed had received the seasonal influenza vaccine. The crude unadjusted overall IVE was 46.1% (95% CI: 13.9–66.3). After adjusting for confounders the overall IVE was 26.0% (95% CI: 0–65.5). In total 211 patients were prescribed an antimicrobial after swab testing. Given a positive influenza POCT result, the odds ratio (OR) of receiving an antiviral was 21.1 (95%CI: 2.4–182.2, p = <0.01) and the OR of being prescribed an antibiotic was 0.6 (95%CI: 0.4–0.9, p = <0.01).

**Discussion:**

Using influenza POCT in a primary care sentinel surveillance network to estimate IVE is feasible and provides comparable results to published IVE estimates. A further advantage is that near patient testing of influenza is associated with improvements in appropriate antiviral and antibiotic use. Larger, randomised studies are needed in primary care to see if these trends are still present and to explore their impact on outcomes.

## Introduction

Influenza is a major cause of clinical and public health burden [1]. It is estimated to account for 11.5% of all episodes of respiratory infection in the UK, with over 50 000 patients requiring hospital admission and at least 2000 deaths per year [2]. Complications are more common in older and younger age groups, over 65 years and under one year respectively [3].

Vaccination is considered as the most effective means for preventing influenza and its complications [4]. A population vaccination coverage target of at least 75% in the elderly population and among risk groups is recommended by the World Health Organization (WHO) [5].

The vaccine requires reformulating annually to match with the characteristics of the circulating influenza viruses which undergo frequent genetic and antigenic changes [6]. Thus influenza vaccine effectiveness (IVE) is assessed annually and observed IVE varies year-to-year [5, 7].

IVE is interpreted as the proportionate reduction in disease among the vaccinated group in real world conditions as opposed to efficacy in ideal conditions, such as a clinical trial [8]. IVE thus varies depending on whether it is measured in secondary or primary care settings [9].

Regulatory agencies such as the European Medicines Agency (EMA) now require vaccine manufacturers to undertake studies in different settings [10]. This includes studies in real world settings such as primary care to provide specific IVE data including product (brand) specific IVE studies as part of their post-licensure requirements, rather than only relying on annual clinical immunogenicity trials of vaccine.

In the last few years rapid molecular point of care test (POCT) platforms for influenza have become widely available in primary care [11]. These highly accurate tests use nucleic acid amplification tests such as reverse transcription polymerase chain reaction (RT-PCR) which had previously been reserved for use in centralised laboratories [12]. This has allowed rapid, accurate pathological confirmation of influenza infection in the community, and is crucial to undertaking IVE studies in primary care.

We have previously shown testing for influenza using rapid molecular POCT machines is feasible in primary care and associated with improvements in appropriate antiviral and antibiotic use. Study practices provided POCT machines performed more tests than other virology sampling practices when their practice population size and respiratory virus infection rates were taken into account. POCT machines also influenced clinical prescribing practices. Patients with a positive influenza POCT test were significantly less likely to be prescribed an antibiotic and significantly more likely to be prescribed antiviral medication (odds ratios of 0.4, 95%CI: 0.19–0.78; and 14.1, 95%CI: 2.85–70.0 respectively) compared to those with a negative influenza POCT result [13].

We hypothesised that the widespread use of influenza POCT in primary care may also contribute to real world evidence on IVE. In this study we use influenza POCT to estimate seasonal IVE for the first time in a primary care sentinel surveillance network. Our secondary aim was to estimate the effect of POCT testing on antimicrobial (antibiotic and antiviral) prescribing.

## Materials and methods

A test-negative case control study was undertaken to estimate the overall and age-specific (6 months-17 years, 18–64 years, ≥65 years old) IVE against medically attended POCT-confirmed influenza.

### Study setting and population

The study took place between October 2019 when influenza viruses was first detected in the sentinel surveillance network and finished early in March 2020 as a result of the national COVID-19 pandemic when the sentinel system moved to a process of remote virology patient self-sampling only [14].

It was nested in the English national influenza surveillance network run by the Oxford-Royal College of General Practitioners (RCGP) Research and Surveillance Centre (RSC) [15]. This is one of the longest established primary care sentinel networks in Europe [16]. The RCGP RSC has been collecting the data used in this study for national surveillance for some years, including providing feedback via dashboards to improve data quality [17]. Previous work has shown that the age and sex distribution of patients in the sentinel network is broadly similar to the English national census distribution [15].

Twelve general practices, from RCGP RSC, with a registered population of 184,813 patients were recruited to the study. Practices were provided with a leaflet explaining the study to staff and eligible patients; and were encouraged to display a poster about the study in their waiting areas. Clinicians in the study practices were encouraged to identify potentially eligible patients aged 6 months and above exhibiting symptoms compatible with influenza-like illness (ILI) and with no contraindication for influenza vaccination for influenza POCT testing.

ILI was defined by the RCGP RSC case definition, which combines key features of the European Centre for Disease Control (ECDC) and WHO definition [18–20].

The Abbott ID Now POCT machine was used for this study. It is a small desktop size POCT machine, that uses an isothermal nucleic acid amplification technology. Its diagnostic accuracy was reported in a systematic review, which reported sensitivity in adults of 80.3% (95% confidence intervals [CI] = 63.7 to 90.8%) and 68.5% (95% CI = 40.2 to 87.2%) for influenza A and B, respectively [11]. Vos et al reported a pooled sensitivity of 81.6% (95% CI = 75.4 to 87.9%) for influenza A and B combined and pooled specificity of 94.0% (95% CI = 86.0 to 100%). This compares with a pooled sensitivity of all rapid molecular test for influenza of 90.9% (95% CI = 88.7 to 93.1%) and pooled specificity of 96.1% (95% CI = 94.2 to 97.9%) [21].

### Ethical statement

Potentially eligible patients over the age of 18 were provided with information about the study by their general practitioner or a study clinician. They were asked for written consent to participate. For potentially eligible children under the age of 18, they were provided with age appropriate information sheet on the study and their parent or legal guardian was asked for written consent for them to participate in the study.

The study was approved by the English National Research Ethics Committees (REC) (Integrated Research Application System ref: 252081; REC ref: 19/WM/0015).

### Data analysis

The primary outcome of interest was POCT virologically-confirmed influenza in the study population.

Patients with POCT virologically-confirmed influenza were defined as cases. Each positive test result was classified by influenza type (A and B) although no information on subtype or lineage was available as part of this study. Controls were deemed as those with ILI who were POCT negative for influenza.

Crude and confounder-adjusted IVE and 95% confidence intervals were estimated using the standard approach based on comparing the odds ratio (OR) of vaccination among influenza-positive study participants with the odds of vaccination among influenza-negative study participants [22].

IVE=(1–OR)x100%.

Confounder-adjusted IVE estimates were derived from logistic regression models. A complete cases analysis was performed as a sensitivity analysis. The following set of covariates were collected from the routine data extracted from the electronic health record and used for confounder adjustment of IVE with backward stepwise elimination to find the best model fit [22].

- Age
- Sex
- Ethnicity, reported in five categories, white, Asian, black, other, or mixed, and maximised using an ontology [23].
- Socio-economic status, measured using the index of multiple deprivation (IMD) [24]. This is a nationally available measure assigned based on post code.
- Any of the following chronic underlying conditions—chronic pulmonary disease, cardiovascular disease, diabetes, liver disease, renal disease, neurologic/neuromuscular conditions, treatment-induced immunosuppression and disease-induced immunosuppression
- Number of GP visits in the 12 months prior to the study period describing a study subject’s healthcare seeking behaviour
- Number of hospitalisations in the 12 months prior to the study period were used as proxy for the severity of the chronic conditions
- Influenza vaccination in previous influenza seasons (at least one)
- Pregnancy
- Use of influenza antivirals
- Pneumococcal vaccination
- Death

Our sample size calculation suggested that we needed at least 100 cases, ideally more to produce an estimate of IVE. This is shown in our protocol [25] and built on our feasibility study of the previous season which did not include calculation of IVE. We calculated the minimal detectable overall VE (1) with 80% power (1 – β) and a two-sided 95% confidence coefficient (1 – α/2) for case-control studies using ‘cases to controls’ ratio of 1:1, 1:2 and 1:4 with the number of cases varying from 100 to 4000, while assuming overall vaccination coverages of 5%, 15%, 30% and 50%.

## Results

In total 648 swabs were taken of which 128 (19.8%) were positive for influenza A or B on swab testing with the POCT machine. Table 1 shows that 263 (40.6%) swabs were taken from males. 66 (10.2%) were aged ≥65 years. 396 (61.1%) were of white ethnicity and 44 (6.3%) were in the most deprived quintile. 175 (27%) swabs were taken from patients who had any underlying risk factors for influenza.

**Table 1:** Characteristics of the patients who had a positive versus negative influenza POCT result.

	All swabs (n = 648)	Influenza +ve (n = 128)	Influenza -ve (n = 520)
**Sex**			
Males	263 (40.6%)	60 (46.9%)	203 (39.0%)
Females	385 (59.4%)	68 (53.1%)	317 (61.0%)
**Age**			
0–17 years	233 (36.0%)	55 (43.0%)	178 (34.2%)
18–64 years	349 (53.8%)	64 (50.0%)	285 (54.8%)
≥65 years	66 (10.2%)	9 (7.0%)	57 (11.0%)
**Ethnicity**			
White ethnicity	396 (61.1%)	76 (59.4%)	320 (61.5%)
All other ethnicity	66 (10.2%)	13 (10.2%)	53 (10.2%)
Ethnicity unknown	186 (28.7%)	39 (30.4%)	147 (28.3%)
**IMD Quintile**			
1 (most deprived)	41 (6.3%)	15 (11.7%)	26 (5.0%)
2	64 (9.9%)	15 (11.7%)	49 (9.4%)
3	120 (18.5%)	27 (21.1%)	93 (17.9%)
4	188 (29.0%)	32 (25.0%)	156 (30.0%)
5 (least deprived)	216 (33.3%)	36 (28.1%)	180 (34.6%)
Unknown	19 (3.0%)	3 (2.4%)	16 (3.1%)
**Risk group**			
Y	175 (27.0%)	28 (21.9%)	147 (28.3%)
N	473 (73.0%)	100 (78.1%)	373 (71.3%)
**Average number of GP visits in last 12 months**	27.6	20.7	29.3
	(95% CI: 25.7–29.5)	(95% CI: 17.1–24.3)	(95% CI: 27.1–31.4)
**Average number hospitalisations in last 12 months**	1.4	1.3	1.4
	(95% CI: 1.3–1.5)	(95% CI: 1.1–1.5)	(95% CI: 1.3–1.5)
**Influenza vaccination in last season**			
Y	181 (27.9%)	27 (21.1%)	154 (29.6%)
N	467 (72.1%)	101 (78.9%)	366 (70.4%)
**Pregnant**			
Y	11 (1.7%)	1 (1%)	10 (2.0%)
N	637 (98.3%)	127 (99%)	510 (98.0%)
**Pneumococcal vaccination**			
Y	177 (27.3%)	31 (24.2%)	146 (28.1%)
N	471 (72.7%)	97 (75.8%)	374 (71.9%)

Approximately three swabs were taken per practice per week over the 19 week duration of the study. The average swabbing rate for POCT practices was 0.4, which compares favourably with our previous influenza POCT study in primary care [13].

### Influenza vaccine effectiveness

193 (29.7%) of those who were swabbed had received the seasonal influenza vaccine. Vaccinated patients were more likely to be older, female, have an underlying risk factor for influenza, have a greater average number of GP visits in last 12 months and more likely to have had pneumococcal vaccination (see Table 2).

**Table 2:** Characteristics of the patients who were sampled and those who had received seasonal influenza vaccination.

	All swabs (n = 648)	Vaccinated patients (n = 193)	Unvaccinated patients (n = 455)
**Sex**			
Males	263 (40.6%)	66 (34.2%)	197 (43.3%)
Females	385 (59.4%)	127 (65.8%)	258 (56.7%)
**Age**			
0–17 years	233 (36.0%)	61 (31.6%)	172 (37.8%)
18–64 years	349 (53.8%)	77 (39.9%)	272 (59.8%)
≥65 years	66 (10.2%)	55 (28.4%)	11 (2.4%)
**Ethnicity**			
White ethnicity	396 (61.1%)	130 (67.4%)	266 (58.5%)
All other ethnicity	66 (10.2%)	20 (10.4%)	46 (10.1%)
Ethnicity unknown	186 (28.7%)	43 (22.2%)	143 (31.4%)
**IMD Quintile**			
1 (most deprived)	41 (6.3%)	9 (4.7%)	32 (7.0%)
2	64 (9.9%)	20 (10.4%)	44 (9.7%)
3	120 (18.5%)	35 (18.1%)	85 (18.7%)
4	188 (29.0%)	56 (29.0%)	132 (29.0%)
5 (least deprived)	216 (33.3%)	71 (36.8%)	145 (31.9%)
Unknown	19 (3.0%)	2 (1.0%)	17 (3.7%)
**Risk group**			
Y	175 (27.0%)	116 (60.1%)	59 (13.0%)
N	473 (73.0%)	77 (39.9%)	396 (87.0%)
**Average number of GP visits in last 12 months**	27.6	41.7	21.6
	(95% CI: 25.7–29.5)	(95% CI: 37.3–46.0)	(95% CI: 19.9–23.3)
**Average number hospitalisations in last 12 months**	1.4	1.6	1.4
	(95% CI: 1.3–1.5)	(95% CI: 1.3–1.8)	(95% CI: 1.2–1.5)
**Influenza vaccination in last season**			
Y	181 (27.9%)	135 (69.9%)	46 (10.1%)
N	467 (72.1%)	58 (30.1%)	409 (89.9%)
**Pregnant**			
Y	11 (1.7%)	7 (3.6%)	4 (1.0%)
N	637 (98.3%)	186 (96.4%)	451 (99.0%)
**Pneumococcal vaccination**			
Y	177 (27.3%)	73 (37.8%)	104 (22.9%)
N	471 (72.7%)	120 (62.2%)	351 (77.1%)

The ratio of the odds of vaccination among influenza-positive study participants compared with the odds of vaccination among influenza-negative study participants was 0.53 (95% CI: 0.34–0.86).

Crude unadjusted overall IVE was 46.1% (95% CI: 13.9–66.3). After adjusting for confounders the overall IVE was 26.0% (95% CI: 0–65.5). Crude unadjusted, age stratified IVE and adjusted, age stratified IVE results are shown in Table 3.

**Table 3:** Age stratified influenza vaccine effectiveness.

Crude age stratified IVE		
**Age groups**	**IVE**	**95% CI**
**0–17 years**	44.7	(0–74.1)
**18–64 years**	46.1	(13.9–66.3)
**≥65 years**	NA	NA
Adjusted, age stratified IVE		
**Age group**	**IVE**	**95% CI**
**0–17 years**	63.5	(0–88.7)
**18–64 years**	24.9	(0–87.2)
**≥65 years**	NA	NA

### Antimicrobial prescribing

Six patients in total were prescribed an antiviral medication in the 7 days following their POCT test. Five (83.3%) had had a positive POCT test for influenza. Table 4 shows the characteristics of those prescribed antivirals versus other patients.

**Table 4:** Characteristics of the patients who were sampled and those who had received antivirals.

	All swabs (n = 648)	Received antivirals (n = 6)	Not received antivirals (n = 642)
**Sex**			
Males	263 (40.6%)	3 (50%)	260 (40.5%)
Females	385 (59.4%)	3 (50%)	382 (59.5%)
**Age**			
0–17 years	233 (36.0%)	1 (16.7%)	232 (36.1%)
18–64 years	349 (53.8%)	5 (83.3%)	344 (53.6%)
≥65 years	66 (10.2%)	0	66 (10.3%)
**Ethnicity**			
White ethnicity	396 (61.1%)	3 (50%)	393 (61.2%)
All other ethnicity	66 (10.2%)	1 (16.7%)	65 (10.1%)
Ethnicity unknown	186 (28.7%)	2 (33.3%)	184 (28.7%)
**IMD Quintile**			
1 (most deprived)	41 (6.3%)	0	41 (6.4%)
2	64 (9.9%)	0	64 (9.9%)
3	120 (18.5%)	4 (66.6%)	116 (18.1%)
4	188 (29.0%)	1 (16.7%)	187 (29.1%)
5 (least deprived)	216 (33.3%)	1 (16.7%)	215 (33.5%)
Unknown	19 (3.0%)	0	19 (3.0%)
**Risk group**			
Y	175 (27.0%)	3 (50%)	172 (26.8%)
N	473 (73.0%)	3 (50%)	470 (73.2%)
**Average number of GP visits in last 12 months**	27.6	28.8	27.5
	(95% CI: 25.7–29.5)	(95% CI: 19.5–38.2)	(95% CI: 25.6–29.5)
**Average number hospitalisations in last 12 months**	1.4	1.3	1.4
	(95% CI: 1.3–1.5)	(95% CI: 0.7–2.0)	(95% CI: 1.3–1.5)
**Influenza vaccination in last season**			
Y	181 (27.9%)	3 (50.0%)	178 (27.7%)
N	467 (72.1%)	3 (50.0%)	464 (72.3%)
**Pregnant**			
Y	11 (1.7%)	0	11 (2.0%)
N	637 (98.3%)	6 (100%)	631 (98.0%)
**Pneumococcal vaccination**			
Y	177 (27.3%)	3 (50.0%)	174 (27.1%)
N	471 (72.7%)	3 (50.0%)	468 (72.9%)

205 patients in total were prescribed an antibiotic medication in the 7 days following their POCT test. 30 (14.6%) had had a positive POCT test for influenza. An antibiotic was more likely to be prescribed for older patients with underlying risk factors for influenza and those who had a history of more visits to the GP in the last 12 months, see Table 5.

**Table 5:** Characteristics of the patients who were sampled and those who had received antibiotics.

	All swabs (n = 648)	Received antibiotics (n = 205)	Not received antibiotics (n = 443)
**Sex**			
Males	263 (40.6%)	82 (40%)	181 (40.9%)
Females	385 (59.4%)	123 (60%)	262 (59.1%)
**Age**			
0–17 years	233 (36.0%)	54 (26.3%)	179 (40.4%)
18–64 years	349 (53.8%)	115 (56.1%)	234 (52.8%)
≥65 years	66 (10.2%)	36 (17.6%)	30 (6.8%)
**Ethnicity**			
White ethnicity	396 (61.1%)	21 (10.2%)	45 (10.2%)
All other ethnicity	66 (10.2%)	127 (62.0%)	269 (60.7%)
Ethnicity unknown	186 (28.7%)	57 (27.8%)	129 (29.1%)
**IMD Quintile**			
1 (most deprived)	41 (6.3%)	14 (6.8%)	27 (6.1%)
2	64 (9.9%)	22 (10.7%)	42 (9.5%)
3	120 (18.5%)	37 (18.0%)	83 (18.7%)
4	188 (29.0%)	60 (29.3%)	128 (28.9%)
5 (least deprived)	216 (33.3%)	68 (33.2%)	148 (33.4%)
Unknown	19 (3.0%)	4 (2.0%)	15 (3.4%)
**Risk group**			
Y	175 (27.0%)	78 (38.0%)	97 (21.9%)
N	473 (73.0%)	127 (62.0%)	346 (78.1%)
**Average number of GP visits in last 12 months**	27.6	33.5	24.82
	(95% CI: 25.7–29.5)	(95% CI: 29.7–37.2)	(95% CI: 22.7–26.9)
**Average number hospitalisations in last 12 months**	1.4	1.5	1.4
	(95% CI: 1.3–1.5)	(95% CI: 1.3–1.7)	(95% CI: 1.2–1.5)
**Influenza vaccination in last season**			
Y	181 (27.9%)	76 (37.1%)	105 (23.7%)
N	467 (72.1%)	129 (62.9%)	338 (76.3%)
**Pregnant**			
Y	11 (1.7%)	4 (2.0%)	7 (1.6%)
N	637 (98.3%)	201 (98.0%)	436 (98.4%)
**Pneumococcal vaccination**			
Y	177 (27.3%)	53 (25.9%)	124 (28.0%)
N	471 (72.7%)	152 (74.1%)	319 (72.0%)

50.7% (*n* = 175/345) of patients received antibiotics following a negative influenza POCT test. The odds of being prescribed an antibiotic given a positive result was 0.6 (95% CI: 0.4–0.9) compared with a negative test.

In comparison, 4% (5/123) of patients received an antiviral following a positive influenza test. The odds of receiving an antiviral given a positive result was 21.1 (95% CI: 2.4–182.2) compared with a negative test.

## Discussion

The is the first time, to our knowledge that IVE has been estimated in a primary care sentinel surveillance using network using POCT machines. We have also confirmed that testing for influenza using POCT machines is associated with improvements in appropriate antimicrobial use.

### Strengths and weakness

The non-randomised design is a limitation of our study, along with its short duration, and small sample size. This was a result of the study having to cease just after halfway through the 2019/2020 influenza season following the COVID-19 pandemic. This resulted in the wide confidence intervals around our IVE and OR point estimates and has restricted our ability to make conclusions from stratified analysis including by influenza virus type and patient demographic characteristics. Additionally, information about influenza subtype was not available from the POCT machines used in this study and restricted interpretation of our results, especially those pertaining to the effectiveness of the seasonal influenza vaccine. Lastly, information about the duration of respiratory illness was not collected before swab testing, thus this limits the conclusions regarding the appropriate use of antiviral medications following POCT.

A strength of the study was that it was conducted amongst practices which were nested in the RCGP RSC English sentinel surveillance network and which had been also been involved in POCT sampling in the previous year. This ensured that practice staff had experience with using the machines, minimising the number of spoiled samples. It also allowed a comparison of the performance of practices using POCT for influenza testing with other practices in the sentinel network that participate in the usual virology sampling programme conducted by Public Health England (PHE).

### Comparison with existing literature

The figures calculated from this study are comparable to the results of published IVE estimates from primary care, including IVE analysis from studies conducted by the ECDC and studies conducted as part of the DRIVE European initiative [26, 27]. This compares with our previous study which found that the odds ratio of receiving an antiviral was 14.1 (95% CI = 2.9 to 70.0, P<0.001) and of receiving an antibiotic was 0.4 (95% CI = 0.2 to 0.8, P = 0.01) given a positive influenza POCT test [13]. Our study reinforces previous findings about the impact of the results of rapid near patient testing for influenza on prescribing practices in non-randomised studies including our own recent study [13, 28].

Given the wide confidence interval of our crude (46.1% (95% CI:13.9–66.3)) and adjusted (26.0% (95% CI: 0–65.5)) IVE, it unsurprisingly fits with other estimates for the same season. IECDC presented estimates from six European studies, covering 10 countries and calculated an IVE between 29% to 61% for all ages in the primary care setting in 2019/20 [26]. The Centre for Disease Control (CDC) in the US calculated the overall IVE of 45% (95% CI = 36%–53%) in the outpatient setting in 2019/20 [29].

For comparison, the DRIVE (Development of Robust and Innovative Vaccine Effectiveness) study undertook a pooled analysis in 2019/20 of data from four primary care sites in Europe using test negative designs that examined 2372 subjects including patients from the UK of which 77 were vaccinated cases. They found the IVE against any influenza in children less than 17y was 64% (95%CI 44–80) for any vaccine [27]. This is comparable with the results from this study where the adjusted IVE against any influenza in children less than 17y was 63.5% (95%CI 0–88.7).

### Implications for research

This study provides the first evidence, to our knowledge, for the use of POCT to estimate IVE within a primary care sentinel network. Further studies however are required with larger sample sizes to ensure that robust point estimates can be obtained for overall and age-specific IVE. Studies that would allow brand/ manufacturer specific IVE estimates to be calculated would also be preferable.

## References

[1] Iuliano A., et al., Estimates of global seasonal influenza-associated respiratory mortality: a modelling study. Lancet, 2018. 391(10127): p. 1285–1300. 10.1016/S0140-6736(17)33293-2 29248255 PMC5935243

[2] Troeger C., et al., Mortality, morbidity, and hospitalisations due to influenza lower respiratory tract infections, 2017: an analysis for the Global Burden of Disease Study 2017. Lancet Respir Med, 2019. 7(1): p. 69–89. 10.1016/S2213-2600(18)30496-X 30553848 PMC6302221

[3] Zhou H., et al., Hospitalizations associated with influenza and respiratory syncytial virus in the United States, 1993–2008. Clin Infect Dis, 2012. 54(10): p. 1427–36. 10.1093/cid/cis211 22495079 PMC3334364

[4] Fact sheet Influenza (Seasonal). 2016; Available from: http://www.who.int/mediacentre/factsheets/fs211/en/.

[5] Osterholm M., et al., Efficacy and effectiveness of influenza vaccines: a systematic review and meta-analysis. Lancet Infect Dis, 2012. 12(1): p. 36–44. 10.1016/S1473-3099(11)70295-X 22032844

[6] Harding A. and Heaton N., Efforts to Improve the Seasonal Influenza Vaccine. Vaccines (Basel), 2018. 6(2). 10.3390/vaccines6020019 29601497 PMC6027170

[7] Ramsay L., et al., The impact of repeated vaccination on influenza vaccine effectiveness: a systematic review and meta-analysis. BMC Med, 2019. 17(1): p. 9. 10.1186/s12916-018-1239-8 30626399 PMC6327561

[8] Lahariya C., Vaccine epidemiology: A review. J Family Med Prim Care, 2016. 5(1): p. 7–15. 10.4103/2249-4863.184616 27453836 PMC4943153

[9] McCartney M., Margaret McCartney: What use is mass flu vaccination? Bmj, 2014. 349: p. g6182. 10.1136/bmj.g6182 25331457 PMC4202689

[10] Guideline on Influenza Vaccines–Non-clinical and Clinical Module. 2016, Eur Med Agency: London.

[11] Merckx J., et al., Diagnostic Accuracy of Novel and Traditional Rapid Tests for Influenza Infection Compared With Reverse Transcriptase Polymerase Chain Reaction: A Systematic Review and Meta-analysis. Ann Intern Med, 2017. 167(6): p. 394–409. 10.7326/M17-0848 28869986

[12] Dziąbowska K., Czaczyk E., and Nidzworski D., Detection Methods of Human and Animal Influenza Virus-Current Trends. Biosensors (Basel), 2018. 8(4). 10.3390/bios8040094 30340339 PMC6315519

[13] de Lusignan S., et al., Integrating molecular point-of-care testing for influenza into primary care: a mixed-methods feasibility study. Br J Gen Pract, 2020. 70(697): p. e555–e562. 10.3399/bjgp20X710897 32661013 PMC7363279

[14] de Lusignan S., et al., Emergence of a Novel Coronavirus (COVID-19): Protocol for Extending Surveillance Used by the Royal College of General Practitioners Research and Surveillance Centre and Public Health England. JMIR Public Health Surveill, 2020. 6(2): p. e18606. 10.2196/18606 32240095 PMC7124955

[15] Correa A., et al., Royal College of General Practitioners Research and Surveillance Centre (RCGP RSC) sentinel network: a cohort profile. BMJ Open., 2016. 6(4): p. e011092. 10.1136/bmjopen-2016-011092 27098827 PMC4838708

[16] de Lusignan S., et al., RCGP Research and Surveillance Centre: 50 years’ surveillance of influenza, infections, and respiratory conditions. Br J Gen Pract, 2017. 67(663): p. 440–441. 10.3399/bjgp17X692645 28963401 PMC5604796

[17] Liyanage H., et al., Near Real Time Feedback of Seasonal Influenza Vaccination and Virological Sampling: Dashboard Utilisation in a Primary Care Sentinel Network. Stud Health Technol Inform, 2020. 270: p. 1339–1340. 10.3233/SHTI200431 32570648

[18] Fitzner J., et al., Revision of clinical case definitions: influenza-like illness and severe acute respiratory infection. Bull World Health Organ, 2018. 96(2): p. 122–128. 10.2471/BLT.17.194514 29403115 PMC5791775

[19] EU case definitions. 03/07/2018 [cited 2020 2th Oct]; Available from: https://www.ecdc.europa.eu/en/surveillance-and-disease-data/eu-case-definitions.

[20] Penttinen P. and Pebody R., Influenza case definitions—optimising sensitivity and specificity. Euro Surveill, 2015. 20(22): p. 21148. 10.2807/1560-7917.es2015.20.22.21148 26062644

[21] Vos L., et al., Rapid Molecular Tests for Influenza, Respiratory Syncytial Virus, and Other Respiratory Viruses: A Systematic Review of Diagnostic Accuracy and Clinical Impact Studies. Clin Infect Dis, 2019. 69(7): p. 1243–1253. 10.1093/cid/ciz056 30689772 PMC7108200

[22] D7.1 Core protocol for type/brand -specific influenza vaccine effectiveness studies (test-negative design studies). 2018; Available from: https://www.drive-eu.org/wp-content/uploads/2018/05/ANNEX1_DRIVE_D7.1_Core-protocol-for-test-negative-design-studies_0.9.pdf.

[23] Tippu Z., et al., Ethnicity Recording in Primary Care Computerised Medical Record Systems: An Ontological Approach. J Innov Health Inform, 2017. 23(4): p. 920. 10.14236/jhi.v23i4.920 28346128

[24] English indices of deprivation 2019. 2019 [cited 2020 29th Oct]; Available from: https://www.gov.uk/government/statistics/english-indices-of-deprivation-2019.

[25] de Lusignan S., et al., Point of care testing for influenza within a national primary care sentinel surveillance network in England: feasibility study (Preprint). JMIR Research Protocols, 2019. 10.2196/14186 PMC6878097 31710303

[26] Rose A., et al., Interim 2019/20 influenza vaccine effectiveness: six European studies, September 2019 to January 2020. Euro Surveill, 2020. 25(10). 10.2807/1560-7917.ES.2020.25.10.2000153 32183932 PMC7078828

[27] Effectiveness, D.o.R.a.I.V., D7.7 Brand-specific influenza vaccine effectiveness in Europe Season 2019/20. 2020.

[28] Lee J., et al., The Clinical Utility of Point-of-Care Tests for Influenza in Ambulatory Care: A Systematic Review and Meta-analysis. Clin Infect Dis, 2019. 69(1): p. 24–33. 10.1093/cid/ciy837 30285232 PMC6579962

[29] Dawood F., et al., Interim Estimates of 2019–20 Seasonal Influenza Vaccine Effectiveness—United States, February 2020. MMWR Morb Mortal Wkly Rep, 2020. 69(7): p. 177–182. 10.15585/mmwr.mm6907a1 32078591 PMC7043386

