# Supplementary material for: Using Point of Care Testing to estimate influenza vaccine effectiveness in the English primary care sentinel surveillance network
Source: PLoS One. 2021 Mar 11;16(3):e0248123. doi: 10.1371/journal.pone.0248123 (PMC7951853; doi:10.1371/journal.pone.0248123)
Supplement: Attachment — Submitted filename: PONE-D-20-39506 - Response to reviewers_20210211.docx [file pone.0248123.s001.docx]

**From:** [em.pone.0.713e8a.ec89bae5@editorialmanager.com](mailto:em.pone.0.713e8a.ec89bae5@editorialmanager.com) <[em.pone.0.713e8a.ec89bae5@editorialmanager.com](mailto:em.pone.0.713e8a.ec89bae5@editorialmanager.com)> on behalf of PLOS ONE <[em@editorialmanager.com](mailto:em@editorialmanager.com)>
**Sent:** Wednesday, February 10, 2021 11:28
**To:** Simon de Lusignan <[simon.delusignan@phc.ox.ac.uk](mailto:simon.delusignan@phc.ox.ac.uk)>
**Subject:** PLOS ONE: Your submission PONE-D-20-39506R1 - [EMID:8cda8bf0fbe23568]

=============================================
IMPORTANT: PLEASE DO NOT REPLY TO THIS EMAIL
If you are unable to complete any points that are requested in this email, please explain why in the "Enter Comments" tab of the online submission form prior to re-submitting your manuscript.  This will enable us to promptly assess your response and progress your manuscript to an Academic Editor at the earliest opportunity.
=============================================

PONE-D-20-39506R1
Using Point of Care Testing to estimate influenza vaccine effectiveness in the English primary care sentinel surveillance network
Prof Simon de Lusignan

Dear Prof de Lusignan,

Thank you for submitting your manuscript entitled "Using Point of Care Testing to estimate influenza vaccine effectiveness in the English primary care sentinel surveillance network" to PLOS ONE.  Your manuscript files have been checked in-house but before we can proceed we need you to address the following issues:

1. Thank you for providing additional details regarding the restrictions on data sharing. In line with our goal of ensuring long-term data availability to all interested researchers, PLOS’ Data Policy states that authors cannot be the sole named individuals responsible for ensuring data access (<http://journals.plos.org/plosone/s/data-availability#loc-acceptable-data-sharing-methods>).

Before we proceed with your manuscript, please also provide contact information for a data access committee (i.e. the University of Oxford Institutional Data Access Committee) to which data requests may be sent. This contact should not be an author.

We will update your Data Availability statement on your behalf to reflect the information you provide.

Oxford reply – We have included in our Data Availability Statement a link below to the website where data requests can be submitted to the University of Oxford Institutional Data Access Committee

<https://orchid.phc.ox.ac.uk/index.php/orchid-data/>

Further enquires about the RCGP RSC network and data requests can also be emailed to the following address

[practiceenquiries@phc.ox.ac.uk](mailto:practiceenquiries@phc.ox.ac.uk)

2. We note that the Introduction, Results, and Discussion sections of your manuscript still contain significant text overlap with the following previously published works:
- <https://www.drive-eu.org/wp-content/uploads/2018/12/DRIVE_D7.1_Core-protocol-for-test-negative-design-studies_1.1.pdf>
-https://eprints.soton.ac.uk/443229/1/BJGP2020.pdf
- <https://www.pharmaceutical-journal.com/news-and-analysis/research-briefing/point-of-care-testing-for-influenza-could-improve-antimicrobial-use-feasibility-study-concludes/20208259.article?firstPass=false>

Oxford reply – The introduction, results and discussion have been substantially revised to remove overlapping text from previous work

Please note that this text overlap must be addressed in order for us to consider your submission further. Please revise the manuscript to eliminate the duplicated text, and - if relevant - provide details as to how the current manuscript advances on previous work.

We will carefully review your manuscript upon resubmission, so please ensure that your revision is thorough.

Your manuscript has been returned to your account. Please log on to PLOS Editorial Manager at <https://www.editorialmanager.com/pone/> to access your manuscript.

Your manuscript can be found in the "Revisions Sent Back to the Author" link under the New Submissions menu. After you have made the changes requested above, please be sure to view and approve the revised PDF after rebuilding the PDF to complete the resubmission process.

Please note that these changes have been requested to comply with submission guidelines and your manuscript will *not* be sent to review until you have fully adhered to our requests.  Once your paper has been seen by an Editor we may return it to you for further information or amendments.

We ask that you address this request within 21 days. If you require additional time, please email the journal office. We are happy to grant extensions of up to one month past this due date. If we have not heard from you within 21 days, your manuscript will be withdrawn from Editorial Manager.

Thank you for submitting your work to PLOS ONE.

Kind regards,

Kirstin Darroch
PLOS ONE

__________________________________________________
In compliance with data protection regulations, you may request that we remove your personal registration details at any time.  (Use the following URL: <https://www.editorialmanager.com/pone/login.asp?a=r>). Please contact the publication office if you have any questions.
